# Holding a country countdown to 2015 conference on Millennium Development Goals (MDGs) – the Zambian experience

**DOI:** 10.1186/1471-2458-14-60

**Published:** 2014-01-21

**Authors:** Victor M Mukonka, Sarai Malumo, Penelope Kalesha, Mary Nambao, Rodgers Mwale, Kasonde Mwinga, Mary Katepa-Bwalya, Olusegan Babaniyi, Elizabeth Mason, Caroline Phiri, Pauline K Wamulume

**Affiliations:** 1 Copperbelt University, School of Medicine, Ndola Central Hospital, 6th Floor, West wing, P. O. Box 71191, Ndola, Zambia; 2 United Nations Population Fund, Country Office, Lusaka, Zambia; 3 Ministry of Community Development, Mother and Child Health, Lusaka, Zambia; 4 United Nations Children’s Fund, Country Office, Lusaka, Zambia; 5 World Health Organisation, Regional Office, Brazzaville, Republic of Congo; 6 World Health Organisation, Country Office, Lusaka, Zambia; 7 World Health Organisation, Headquarters, Geneva, Switzerland; 8 Ministry of Health, National Malaria Control Centre, P. O. Box 32509, Lusaka, Zambia

**Keywords:** Country countdown, Maternal, Newborn and child health, Millennium Development Goals

## Abstract

Initiatives such as the Country Countdown to 2015 Conference on Millennium Development Goals (MDGs) have provided countries with high maternal and child deaths like Zambia a platform to assess progress, discuss challenges and share lessons learnt as a conduit for national commitment to reaching and attaining the MDGs four and five. This paper discusses and highlights the process of holding a successful country countdown conference and shares Zambia’s experience with other countries planning to organise country countdown to 2015 Conferences on MDGs.

## Correspondence

### Background

In 2000, the United Nations Millennium Declaration committed governments and the international community to address major issues related to peace, development and human rights. Millennium Development Goals (MDGs) Countdown to 2015 is a global initiative that tracks global and national progress towards the achievement of MDGs four and five; focuses on coverage of effective interventions for maternal, newborn and child survival and reports on 75 priority countries which account for more than 95% of maternal and child mortality worldwide [1].

Zambia held its first ever Countdown to 2015 Conference on Maternal, Newborn and Child Health (MNCH) in 2008. The conference was meant to raise awareness, take stock of achievements and bottle necks, identify areas for improvement in programming and budgeting, strengthen monitoring, track progress and build a common vision and strategies towards the attainment of the MDGs. The event also facilitated identification of gaps in actions and knowledge that hinder progress and building consensus on defined roles and specific actions for Zambia to achieve the health-related MDGs. The Ministry of Health engaged key stakeholders and partners to participate in planning and holding of the national conference [2].

Zambia is among countries in the world with very high under five mortality rate at 119 per 1,000 live births and maternal mortality ratio at 591 per 100,000 live births [3].

The objectives of the countdown conference were mainly to share information on the global Countdown to 2015 Conference for MNCH, allow national and sub-national stakeholders to take stock of Zambia’s progress towards meeting health-related MDGs, coverage of high impact interventions and re-positioning of priorities with focus on what needed to be done to stimulate country actions [2].

### Process

Zambia decided to hold a Country Countdown to 2015 Conference on MDGs following the country’s participation in the first Countdown to 2015 Conference held in London from 13th to 14th December in 2005 whose theme focused on tracking progress in child survival. The Conference followed a stringent step-by- step process which included the following:

1. Planning

••Formation of a main committee supported by sub-committees with specific tasks and holding of regular briefing meetings.

••Development and sharing of a detailed and costed work plan and budget with all key stakeholders.

••Development of an information package for participants, media and decision-makers.

••Identification of lead partners to spearhead agreed upon tasks.

2. Data content and analysis

••Collection, analysis and disaggregation of data and development of profiles for both national and sub-national levels.

••Consolidation of national profile which included new data from national and global reports.

3. Media engagement

••Inclusion of media practitioners in sub-committees, orientation and holding of regular media briefings for Journalists.

••Placement and broadcast of various media articles and programmes including press coverage of the conference.

4. Countdown Conference

The Minister of Health officially opened the countdown conference. The World Health Organisation Regional Office for Africa Director of Reproductive and Family Health presented a keynote speech. Participants included ministers from line- ministries, country representatives of various United Nations (UN) agencies and cooperating partners, policy makers, implementers, researchers, professional bodies, academics, parliamentarians, media, Non Governmental Organisations and the private sector.

### Presentations

Twenty five presentations were given which addressed country-level coverage and trends of proven high impact interventions to reduce maternal, newborn and child mortality, health system, financing and equity. The presentations described district profiles, thus offering provincial and district participants an opportunity for information sharing and peer-to-peer learning. The conference also promoted competition and stimulated districts to improve performance.

Zambia has made important advances in the last five years as a result of the country countdown conference. The most significant actions and direct results of the countdown conference at country and global levels include:

### Country level

1. Creation of a separate budget line for reproductive health and commodities in the national budget in 2009.

2. Introduction of direct funding to training institutions, commencement of direct midwifery training, expansion and rehabilitation of training institutions to increase health professionals in 2009.

3. Institutionalisation of maternal deaths reviews in all districts in 2009.

4. Amendment of the legislation authorising midwives to administer a set of core interventions to reduce maternal and child mortality in 2010.

5. Launch of Campaign on Accelerated Reduction of Maternal Mortality in Africa (CARMMA) in 2010.

6. Introduction of the safe motherhood week and mandatory inclusion of MNCH activities in district action plans in 2010.

7. Development of the MNCH road map in 2013.

### Global level

1. The UN System and the global partnership for MNCH invited Zambia to share experiences at the following global events.

••Women Deliver Conference in Washington held on June 13th 2012.

••2012 Annual African Women Parliamentarians Conference, Johannesburg, South Africa from 3rd to 4th October, 2012.

••African Union International Conference on MNCH in Africa held in Johannesburg, South Africa from 1st to 3rd August, 2013.

••Women Deliver Conference in Kuala Lumpur, Malaysia from 28th to 30th May, 2013.

2. Participation in the development of countdown tools for the MNCH Partnership.

3. Provision of technical support and serve as a resource base for countries in the region planning to conduct countdown conferences.

Other countries which have held country countdowns are Nigeria and Senegal with the following outcomes respectively:

Nigeria embarked on an Integrated MNCH Strategy and regularly produces up-to-date child and maternal health profiles for each of its 36 states, modelled on the Countdown country profiles. These sub-national profiles have led to increased commitments by the government and its partners [4].

Senegal’s national Countdown event led to a number of new commitments including the development of a new plan for child survival to complement the existing national Roadmap for Maternal Health [4].

### Key lessons learnt

1. Strong leadership at senior level in the Ministry of Health is important.

2. High-level ownership by government through the Ministry of Health is critical to ensure partner support and participation.

3. Planning for the conference must commence in good time.

4. Costed detailed workplan and budget is a priority and should be shared with all partners and follow up on pledges made to support the conference.

5. Planning and holding of the conference should be inclusive and consultative.

6. Countdown conferences must be budgeted for in national budgets.

7. Partners must include countdown conferences in their work plans.

8. Decision-makers should be represented in the main and sub-committees to ensure fulfilment of commitments.

9. The media should be involved throughout the whole planning and implementation process.

## Conclusion

Countries with high maternal and child mortality rates are encouraged to plan and conduct countdown conferences and ensure adequate preparations are undertaken involving all key stakeholders.

The countdown conference should be considered as part of a country accountability framework and used to assess national, sub-national and district progress. The countdown conference is a powerful tool and in-country mechanism that can be utilised to take stock of achievements and bottlenecks, identify areas and population groups that require attention, monitor progress towards meeting health-related MDGs and stimulate country actions.

The Zambian experience has demonstrated that the countdown conference assisted the country in repositioning priorities and re-focusing of required actions to achieve maximum impact. The conference also assisted in mobilising additional resources through creation of a separate budget line for MNCH services, equipment and commodities in the national budget.

## Competing interests

The authors declare that they have no competing interests.

## Authors’ contributions

VMM having spearheaded the country countdown conference, conceived the idea, coordinated and prepared the technical framework and drafted the article. PKW, SM, MN, RM, MKB, KM, PK, CP, provided technical input, participated in sequencing alignment of the technical framework and reviewed the article. OB, EM contributed to the draft and evaluated the article. All authors read and approved the article.

## Pre-publication history

The pre-publication history for this paper can be accessed here:

http://www.biomedcentral.com/1471-2458/14/60/prepub
